# A smartphone application to enhance bowel preparation for first-time colonoscopy: a randomized controlled study

**DOI:** 10.3389/fmed.2024.1376586

**Published:** 2024-04-24

**Authors:** Tanawat Pattarapuntakul, Thanyaporn Kanchanasuwan, Apichat Kaewdech, Thanawin Wong, Nisa Netinatsunton, Nalerdon Chalermsuksant, Pimsiri Sripongpun

**Affiliations:** ^1^Gastroenterology and Hepatology Unit, Division of Internal Medicine, Faculty of Medicine, Prince of Songkla University, Hat Yai, Songkhla, Thailand; ^2^Nantana-Kriangkrai Chotiwattanaphan Institute of Gastroenterology and Hepatology, Faculty of Medicine, Prince of Songkla University, Hat Yai, Songkhla, Thailand; ^3^Gastroenterology and Hepatology Unit, Division of Internal Medicine, Koh Samui Hospital, Suratthani, Thailand; ^4^Division of Gastroenterology and Hepatology, Department of Internal Medicine, Faculty of Medicine, Vajira Hospital, Navamindradhiraj University, Bangkok, Thailand

**Keywords:** colonoscopy, smartphone application, colorectal cancer, adenoma detection rate, bowel preparation scale score

## Abstract

**Background and aims:**

Colonoscopy is an essential cancer screening tool; however, bowel preparation is a multifaceted process that involves several steps. Proper preparation is crucial for a successful colonoscopy in terms of diagnostic accuracy and procedural safety. We evaluated the performance of a smartphone application with bowel preparation instructions on individuals undergoing their first colonoscopy.

**Methods:**

In this randomized, prospective, endoscopist-blinded study, participants were scheduled to undergo their first colonoscopy between January 2020 and January 2022. The study protocol was registered at Thai Clinical Trials Registry (TCTR20190928002). They were randomly assigned to the smartphone education application (APP) or the standard education (control) group. The Boston Bowel Preparation Scale (BBPS) score, polyp detection rate (PDR), and adenoma detection rate (ADR) were compared. Factors associated with excellent bowel preparation were also evaluated.

**Results:**

In total, 119 patients (APP group, *n* = 57; control group, *n* = 62) underwent their first colonoscopy. The mean BBPS score and proportion of excellent bowel preparation (BBPS≥8) were significantly higher in the APP group than in the control group. Smartphone application-guided bowel preparation achieved a higher proportion of adequate and excellent bowel preparation scores, was associated with other quality indicators, and achieved the target ADR, cecal intubation rate, and adequate withdrawal time.

**Conclusion:**

This application may be a user-friendly option to improve the first-time colonoscopy experience, resulting in effective screening of colorectal cancer.

**Clinical trial registration:**

The study protocol was registered at Thai Clinical Trials Registry (TCTR20190928002).

## Introduction

Colonoscopy is an effective tool for the screening and surveillance of colorectal cancer (CRC), which can reduce cancer-related mortality worldwide (1). Identification and removal of neoplastic polyps are key factors in CRC prevention. The quality of bowel preparation is crucial for successful colonoscopy in terms of diagnostic accuracy and procedural safety (2, 3). The adequacy of bowel preparation is affected by numerous factors, including bowel cleanliness, scoring of the colonic bubble, patient education, and compliance with instructions regarding purgatives and diet restrictions (2, 4–7).

Approximately 20–25% of patients have been reported to have inadequate bowel preparation, and the number could be as high as 50% in those with chronic intestinal disease, e.g., inflammatory bowel disease (IBD), resulting in unfavorable consequences, such as missed adenomas, risk of procedure-related adverse events, a higher rate of repeat colonoscopy, and increased overall costs (8–10). Bowel preparation is a multifaceted process and involves multiple steps, including instructions for purgative use, dietary restriction, medical compliance, and adverse events. Patient satisfaction also affects bowel cleanliness. Hence, the Quality Committee of the European Society of Gastrointestinal Endoscopy recommends a minimum of 90% adequate bowel preparation (measured using validated scales) for standard colonoscopy (4, 11).

Patient education on bowel cleansing using written instructions and verbal explanations is widely used. However, owing to the rapidly increasing number of individuals who use smartphones daily, studies have demonstrated the efficacy of educating individuals about bowel cleansing through mobile health technologies, such as automated short message services, video clips, and smartphone applications (12–19). There is an interest in smartphone applications to provide information and facilitate patient communication. This is achieved by displaying text instructions, videos, dietary restriction programs, time notifications to begin bowel preparation, and time notifications for the second dose of purgative agents to enhance bowel quality.

This study aimed to evaluate the performance of smartphone application to reinforce bowel preparation education in patients undergoing their first colonoscopy. We also aimed to compare bowel preparation scores, polyp detection rates (PDRs), and adenoma detection rates (ADRs) between patients who used the smartphone application and a control group.

## Methods

### Study design

We conducted a prospective, endoscopist-blinded, single-center, randomized controlled study. Patients who regularly used smartphones in their daily lives and were scheduled to undergo their first colonoscopy between January 2020 and January 2022 were invited to participate in the study. All endoscopic procedures were performed at Songklanagarind Hospital, the only tertiary care university hospital in Southern Thailand. Patients aged 18–70 years scheduled for their first elective colonoscopy and had been regularly used smartphones were included. The exclusion criteria were as follows: (1) history of abdominal surgery; (2) severe comorbid disease; (3) American Society of Anaesthesiologists risk class III or higher; (4) severe mental illness; (5) known inflammatory bowel disease; (6) active gastrointestinal bleeding; (7) history of gastrointestinal malignancy; (8) allergy to purgative agents; and (9) pregnancy. Eligible participants were randomly assigned (1,1) to either the smartphone application group (APP group) or the conventional method group (control group). Randomization was performed using a computer-generated sequence in a block of four, and the study groups were blinded by sealing consecutively numbered envelopes within the sealed box. The study protocol was approved by the Ethics Committee of the Faculty of Medicine, Prince of Songkla University (REC No. 62-360-14-3) and registered at Thai Clinical Trials Registry (Thaiclinicaltrials.org: TCTR20190928002). Written informed consent was obtained from all participants before their enrolment in the study.

### Instructions on bowel preparation

For the standard regimen of bowel preparation purgatives, split dosing of polyethylene glycol electrolyte powder (PEG-ELYTE, a hospital-prepared solution, contains 188 grams of polyethylene glycon 4000, 2.93 grams of sodium chloride, 3.37 grams of sodium bicarbonate, 11.37 grams of sodium sulfate, and 1.485 grams of potassium chloride. Each unit of PEG-ELYTE should be dissolved in 2000 mL of sterile water for use) was utilized. The first dose was administered at night, with 2000 mL divided into 250 mL portions to be taken every 15 min until the entire amount was consumed within 2 h. The administration of the second dose was scheduled for early consumption on the day of the colonoscopy. Prior to the procedure, all patients were advised to consume a low-fiber diet three days in advance and allowed to drink clear fluid until 4 h prior to the procedure.

Patients in the control group received written instructions and conventional verbal explanations. The nursing team provided a detailed explanation of the procedural approach, the specific time intervals for delivering the purgative agent and medicine, as well as the importance of adhering to a low-fiber diet (avoiding vegetables, fruits, nuts, and grains) for three days prior to the colonoscopy. Additionally, they highlighted the potential adverse effects associated with the procedure.

**Figure 1:**
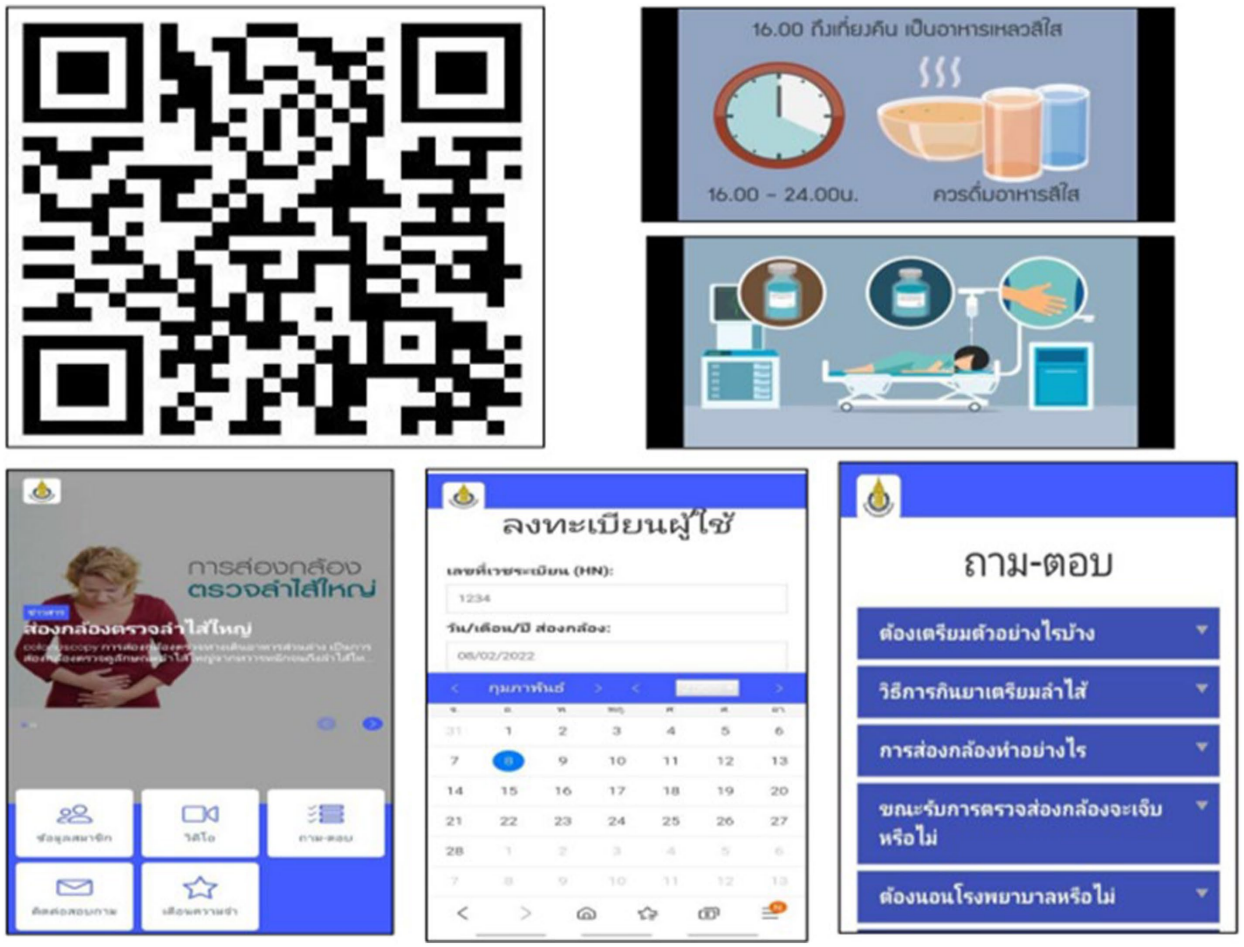
Examples of application screen captures: A QR code for downloading and registering the smartphone application (iOS and Android). Application information included animated video clips, frequently asked questions and answers, protocol notifications, and endoscopic center contact information (Thai version).

In the APP group, patients were required to download the study application on their iOS or Android mobile phones. The application is available at no cost and comprises four distinct sections, with all educational components presented in the Thai language. The first part was a three-minute animated video clip that provided content similar to the written instructions, including the procedural method, the intervals for administering the purgative agent and medication, diet restrictions before colonoscopy, and possible procedure-related adverse events. The second part was a pop-up listing frequently asked questions and answers. The third part was a notification message sent to the patients three days before the colonoscopy, reminding them about a low-fiber diet and one day before the colonoscopy to remind them to take the first dose of the purgative agent. The fourth part included the contact information of the endoscopy center nursing staff for any enquiries. Screen-capture examples from this application are shown in Figure 1. All participants received an official instruction leaflet from the endoscopy center.

All colonoscopy procedures were scheduled by endoscopists who performed at least 300 colonoscopies per year. All procedures were performed between 9:00 a.m. and 1:00 p.m. every weekday.

### Data collection and colonoscopy procedure

All patients were interviewed on the day of the colonoscopy by an endoscopic nurse who was unaware of the study group. The participants finished a bowel cleanliness questionnaire that inquired about the frequency of bowel movements and characteristics of their most recent bowel movement prior to the procedure. To provide this information, they selected an image from the questionnaire that best represented their last stool. Information regarding the start time, completeness of purgative agent administration, and adverse events during bowel preparation, such as vomiting, abdominal pain, and urticarial rash, if occurred, were also recorded.

Colonoscopies were performed by endoscopists who were blinded to the allocation arms. The procedure employed a colonoscope (CF-H190L/I or PCF-H190DL; Olympus Optical Co., Ltd., Tokyo, Japan). Intraprocedural information, including insertion time, cecal intubation rate, withdrawal time, PDR, ADR, Boston bowel preparation scale score (BBPS), and adverse events, was collected.

Bowel preparation was assessed using the BBPS (20), in which endoscopists evaluated the colon in three segments: right (caecum and ascending colon), transverse (hepatic and splenic flexures), and left (descending colon, sigmoid, and rectum). Each segment was scored on a numerical scale ranging from 0 to 3. The three-segment scores were added to obtain a total score ranging from 0 (unprepared colon) to 9 (entirely clean colon). If the procedure was aborted owing to inadequate bowel preparation, the non-visualized proximal segments were assigned a score of 0.

We assessed the polyp by the narrow band imaging technology for predicting the histology of the polyp under NBI International Colorectal Endoscopic (NICE) classification (21) by the experienced discrete endoscopists.

A polyp with a size >5 mm was removed if detected during colonoscopy. Hot snare polypectomy with or without submucosal injection (saline-assisted polypectomy) or cold snare polypectomy was performed at the discretion of the endoscopists. Cold snare polypectomy or cold biopsy polypectomy was performed for polyps <5 mm. Gastrointestinal pathologists performed the histopathological assessments of all resected polyps. The PDR, ADR, and advanced adenoma detection rate (aADR) were recorded.

### Outcome measurements

The primary endpoint of this study was the adequacy of bowel preparation, which was assessed using the BBPS. Adequate bowel preparation was defined as a total BBPS score of ≥6 and ≥ 2 in each colonic segment. A BBPS score of ≥8 indicated excellent bowel preparation (22). The secondary endpoints were colonoscopy withdrawal time, cecal intubation rate, PDR, ADR, aADR, adverse events during bowel preparation.

### Statistical analysis

Descriptive statistics were used; continuous data were expressed as mean ± standard deviation or median with interquartile range according to data distribution, and categorical data were presented as the number of participants (%). For the comparisons of the outcomes between the APP group and the control group, Fisher’s exact and rank-sum tests were performed for categorical data, e.g., PDR, ADR, and the proportion of patients with excellent bowel preparation. The *t*-test and Wilcoxson-ranksum test were used for the comparisons of continuous variables such as withdrawal time and BBBP between the two groups, as appropriate. *p*-values of <0.05 were considered statistically significant. All statistical analyses were performed using the R software version 4.2.1 (Vienna, Austria).

## Results

### Patient characteristics

**Figure 2:**
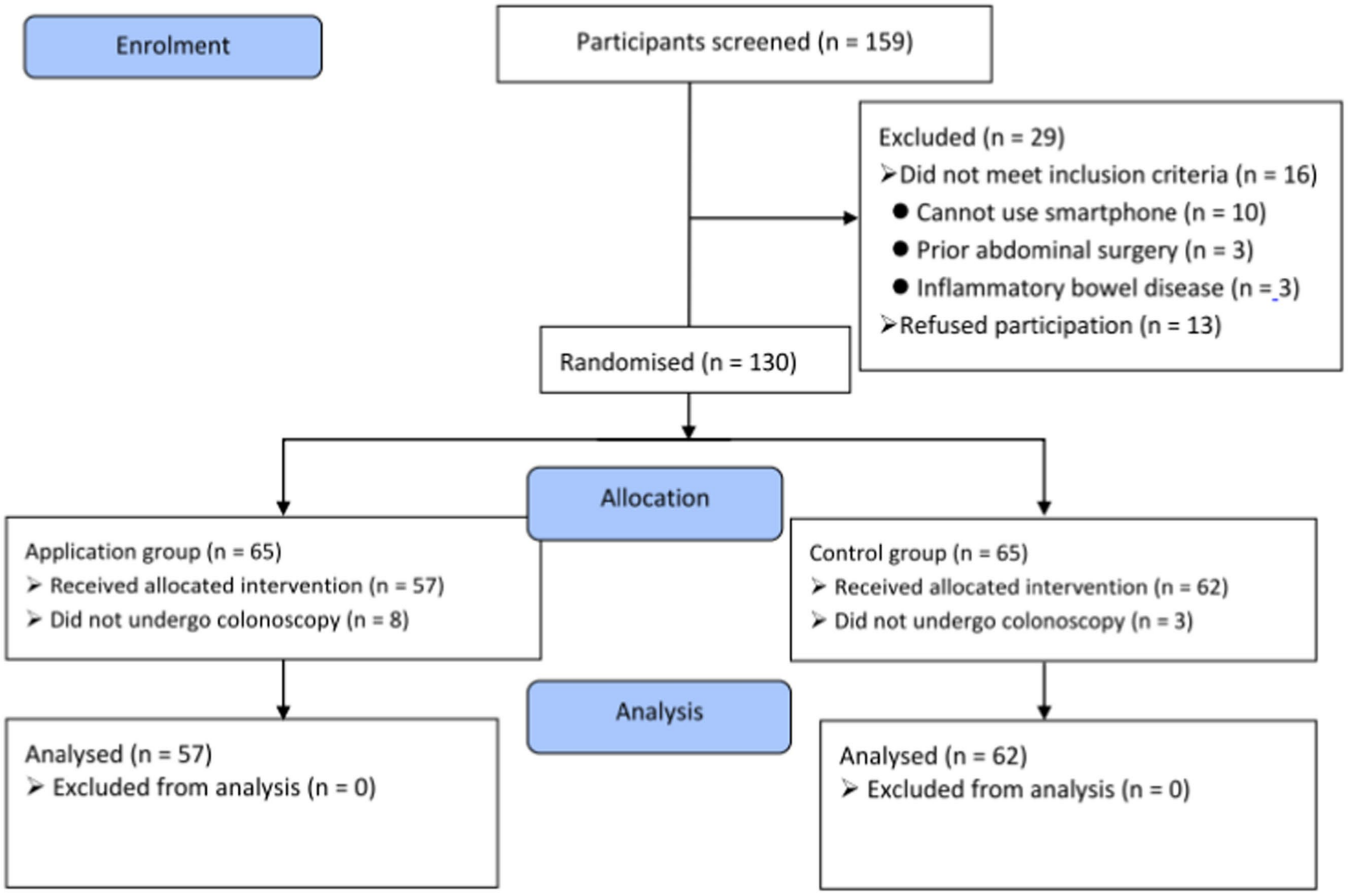
Flow chart of the study.

One hundred and fifty-nine participants were screened for this study. Subsequently, 29 patients were excluded for various reasons (Figure 2). The 130 eligible patients were enrolled and randomized equally into the study groups. Sixty-five patients were randomized into the smartphone education application (APP) group and scheduled for elective colonoscopy; however, eight patients could not undergo colonoscopy on the scheduled date because of coronavirus disease 2019 (COVID-19) and caregiver problems. Another 65 patients were assigned to the control group, three of whom did not undergo the procedure because of COVID-19. Hence, a total of 119 patients were analyzed (57 and 62 in the APP and control groups, respectively).

The patients’ baseline characteristics were comparable between the two groups, except for a higher proportion of patients in the control group with comorbid diseases such as hypertension, dyslipidaemia, and chronic liver disease. Moreover, patients in the APP group used the YouTube application more frequently in their daily lives (Table 1) than the control group. The primary indication for colonoscopy was CRC screening (60.5%), followed by changes in bowel habit (14.3%).

**Table 1:** Patients’ baseline characteristics.

Variables	APP group (*n* = 57)	Control group (*n* = 62)	*p*-value
Female sex^#^	32 (56.1)	32 (53.8)	0.756
Age (years)*	55.7 ± 9.5	57.7 ± 9.8	0.259
Body mass index (kg/m^2^)*	24.1 ± 3.7	23.7 ± 3.5	0.564
Comorbid disease^#^			
Diabetic mellitus	9 (15.8)	13 (21)	0.624
Hypertension	9 (15.8)	21 (33.9)	0.04
Dyslipidemia	2 (3.5)	14 (22.6)	0.05
Cardiovascular disease	4 (7)	6 (9.7)	0.745
Chronic liver disease	3 (5.3)	12 (19.4)	0.042
Chronic kidney disease	3 (5.3)	2 (3.2)	0.67
Need caregiver^#^	15 (26.3)	20 (32.3)	0.611
Smartphone application use daily^#^			
Facebook application	38 (66.7)	38 (61.3)	0.675
YouTube application	17 (29.8)	8 (12.9)	0.042
LINE, a messaging application	57 (100)	62 (100)	0.647
Indication for colonoscopy^#^			
CRC screening	36 (63.2)	36 (58.1)	0.704
Chronic abdominal pain	4 (7)	4 (6.5)	1
Bowel habit change	6 (10.5)	11 (17.7)	0.389
Chronic constipation	6 (10.5)	1 (1.6)	0.054
Iron deficiency anemia	3 (5.3)	8 (12.9)	0.262
Lower gastrointestinal bleeding	2 (3.5)	2 (3.2)	1

^*^Data are expressed as mean ± SD, ^#^Data are expressed as *n* (%). APP group, the smartphone application group; CRC, colorectal cancer.

### Bowel preparation outcomes

**Figure 3:**
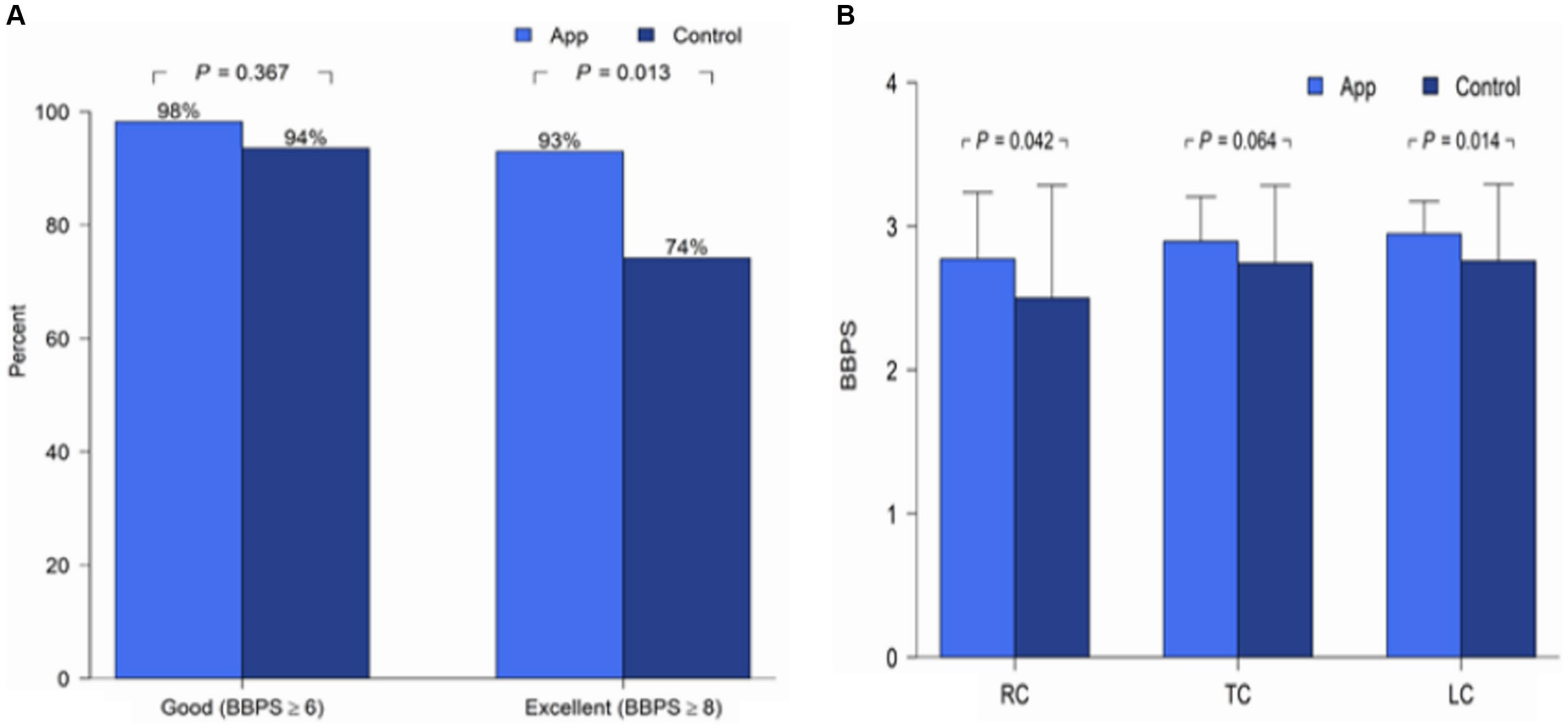
**(A)** Adequate and excellent BBPS scores between the groups. **(B)** Mean BBPS score in each colonic segment between groups. BBPS, Boston Bowel Preparation Scale; RC, Right colon; TC, Transverse colon; LC, Left colon.

The main outcome of BBPS was compared between the two groups, as shown in Table 2. Given the non-normal distribution of BBPS scores in our study, we presented the median values (IQR). Nevertheless, in previous studies addressing the same topic, the authors typically presented the mean BBPS values. Therefore, we also provide these numbers as well. The BBPS in the APP group was significantly higher than that in the control group. The proportion of patients with adequate bowel preparation (BBPS ≥6) was numerically higher in the APP group than in the control group (98.2% vs. 93.5%, *p* = 0.367). And a significantly higher proportion of patients with excellent bowel preparation (BBPS ≥8) was observed in the APP group than in the control group (93% vs. 74.2%, *p* = 0.013) (Figure 3A). Further analyses of each colonic segment revealed that the mean BBPS scores were higher in the APP group than in the control group for every colonic segment (right colon: 2.8 vs. 2.5, *p* = 0.042; transverse colon: 2.9 vs. 2.7, *p* = 0.064; left colon: 2.9 vs. 2.8, *p* = 0.014; Figure 3B).

**Table 2:** BBPS outcomes and polyp detection rate.

Colonoscopy outcomes	Application group (*n* = 57)	Control (*n* = 62)	*P*-value
Quality of bowel preparation			
BBPS, mean (SD)	8.6 (0.8)	8 (1.6)	0.013
BBPS, median (IQR)	9 (9,9)	9 (7.25,9)	0.021
Adequate bowel preparation^#^			0.367
Good, BBPS ≥6	56 (98.2)	58 (93.5)	
Insufficient, BBPS <6	1 (1.8)	4 (6.5)	
Excellent bowel preparation^#^			0.012
Excellent, BBPS ≥8	53 (93)	46 (74.2)	
BBPS for colonic segments, mean (SD) *			
Right side colon	2.8 (0.5)	2.5 (0.7)	0.042
Transverse colon	2.9 (0.3)	2.7 (0.5)	0.064
Left side colon	2.9 (0.2)	2.8 (0.5)	0.014
BBPS for colonic segments, median (IQR)^†^			
Right side colon	3 (3,3)	3 (2,3)	0.043
Transverse colon	3 (3,3)	3 (3,3)	0.077
Left side colon	3 (3,3)	3 (3,3)	0.019
Cecal intubation^#^	57 (100)	58 (93.5)	0.12
Withdrawal time, min^†^	9.3 (7.5, 12)	11.2 (8.5, 15.9)	0.034
Total procedure time, min^†^	21.3 (14, 35)	27.5 (19.2,38.8)	0.073
Polyp detection^#^			
Polyp detection rate	25 (43.9)	25 (40.3)	0.838
Adenoma detection rate	16 (28.1)	17 (27.4)	1
Advanced adenoma detection rate	2 (3.5)	3 (4.8)	1
Adenocarcinoma	2 (3.5)	9 (14.5)	0.079

*Data are expressed as mean + SD, ^†^Data are expressed as median (IQR), ^#^Data are expressed as *n* (%). BBPS, Boston Bowel Preparation Scale; SD, Standard deviation; IQR, Interquartile range.

### Quality indicator outcomes

The cecal intubation rate was slightly higher in the APP group than in the control group (100% vs. 93.5%, *p* = 0.12), and the mean withdrawal time was >9 min in both groups. Interestingly, a longer mean withdrawal time was observed in the control group compared to the APP group (15.1 ± 11.9 min vs. 10.9 ± 5.9 min, *p* = 0.018).

### Polyp detection outcomes

**Figure 4:**
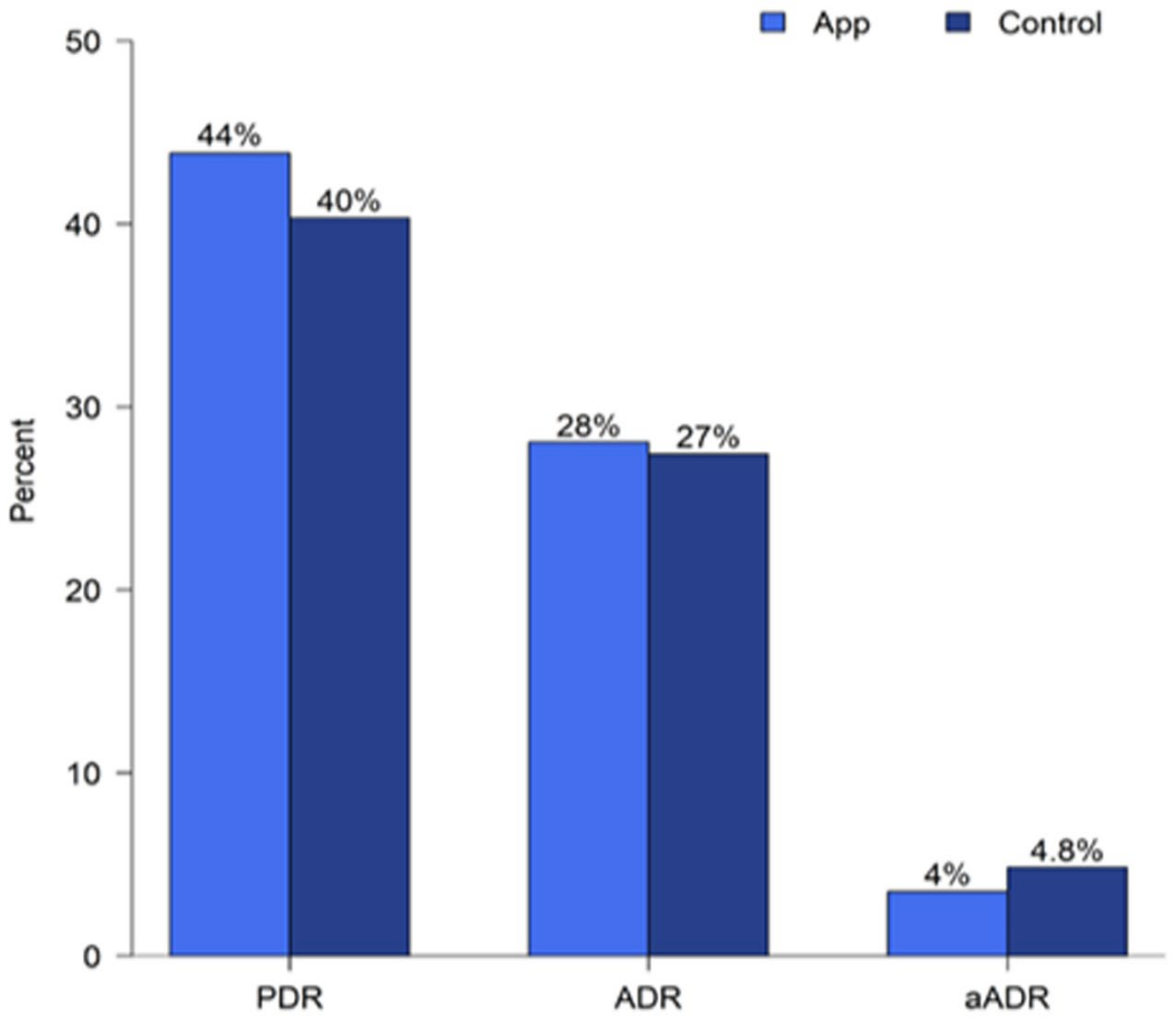
The polyp detection rate (PDR), adenoma detection rate (ADR), and advanced adenoma detection rate (aADR) between groups.

The two groups showed no significant differences in the PDR, ADR, or aADR (Table 2; Figure 4). However, slightly higher proportions of patients with PDR and ADR were observed in the APP group than in the control group.

## Discussion

High-quality colonoscopy enhances outcomes by providing satisfactory colonic visualization and aiding in polyp detection. Bowel preparation is essential in determining colonoscopy quality; however, it is a multistep task. The split-dose PEG-based regimen showed 20–25% inadequate bowel preparation in general, and even higher in those with IBD (9, 23, 24). Therefore, effective preparation guidance is clinically essential. The present study showed that using a smartphone application to deliver bowel preparation instructions favorably affects bowel cleanliness outcomes. The APP group had a significantly higher rate of excellent bowel cleanliness and required a shorter withdrawal time, whereas the PDR and ADR were comparable to those of the control group.

Regarding technology-assisted bowel preparation education, various techniques, such as educational video aids, smartphone applications, and video clips sent via short messaging services, have been reported to be more effective than standard verbal instructions regarding adequate bowel preparation (13–19, 25, 26). Walter et al. published a randomized controlled trial (RCT) involving 500 patients in 2021 using a technique similar to that in our study. They found that smartphone education for bowel preparation instruction had a higher rate of adequate bowel preparation than standard education (92.3% vs. 83.1%, *p* = 0.0023) (18). A meta-analysis published in 2022 also demonstrated the benefit of smartphone-assisted education in bowel preparation, with the pooled risk ratio of achieving adequate bowel preparation of 1.15 (95%CI 1.07–1.23) over the control group (19). Nonetheless, results from subsequent RCTs found no significant difference in the proportion of adequate bowel preparation between the two groups (27, 28). However, Our data showed a significantly better bowel cleanliness represented by the better BBPS in the APP group but the proportion of patients achieving adequate bowel preparation was not significantly different. It is come to our attention that the dissimilarity between the adequate bowel preparation outcome of the present study and others is not due to the ineffectiveness of the smartphone application but the higher rate of adequate bowel preparation in the control group, which was as high as 93%. The higher adequate bowel cleanliness rate in our study may be attributable to the dual mode of instruction, verbal instruction by nursing staff and instructional leaflets, and the Thai culture, in which a family member or caregiver accompanies the patient during almost every hospital visit. These individuals can also help reminding patients about the preparation steps, resulting in a higher rate of adequate bowel cleanliness over 90% in both groups.

Our study showed a significantly higher rate of excellent bowel preparation in the APP group than in the control group. This confirms the performance of the smartphone application for bowel preparation. The longer interval between verbal instruction and the date of the colonoscopy appointment is an intriguing variable because verbal information can easily be forgotten over time (29). Smartphone application instructions may be advantageous and can overcome the problem mentioned above, as they can be rewatched. The notifications help remind patients to start the three-day low-fiber diet and the time to begin administering a purgative agent.

The right side of the colon is usually a hidden area of concern regarding bowel cleanliness because important polyps may be missed in this area (30, 31). Interestingly, our data showed that the APP group had a significantly higher mean BBPS score in the right colon than the control group, which might benefit the visualization and detection of right-sided colonic lesions. We also demonstrated other quality indicators of colonoscopy; for example, the cecal intubation rate and the mean withdrawal time were > 90% and > 9 min in both groups, respectively. However, the score of colonic bubble and the use of simethicone were not systematically recorded in our practice.

ADR is currently accepted as an indicator of colonoscopy quality. Adequate bowel cleanliness improves bowel visualization, especially on the right side of the colon, and increases PDR and ADR (29, 32). In the present study, both groups had high PDR and ADR. This is consistent with the comparable adequate bowel preparation rates between the two groups.

Our study has several strengths. This is a randomized controlled study in which the assessors (endoscopists) were blinded to the assigned group of patients, providing solid objective evidence. The application is available in the local language and can be downloaded at no cost from both iOS and Android devices. Our study confirmed the applicability of smartphone applications for the first-time experienced bowel preparation in patients with different indications for colonoscopy. Also, In addition, this study was conducted during the COVID-19 pandemic, which resulted in reduced time, fewer verbal instructions, and less on-site contact.

However, this study has some limitations. Our study was conducted at a single-center university hospital, in which the participants may have had more favorable profiles, such as regular use of smartphones, more internet accessibility, and higher socioeconomic status, than the general population. Also, the application was available on in Thai language version. The generalizability of the current version of application may be limited. Nonetheless, it confirms the concept that using smartphone-assisted bowel preparation is beneficial in patients undergoing colonoscopy from various regions including Thailand where the English is not the official language. Additionally, we included only patients underwent their first-time colonoscopy to minimize the risk of bias from prior bowel preparation knowledge in those who had repeated colonoscopy and excluding patients with known chronic intestinal disease. Whether the application will benefit those who need repeated colonoscopy but with troublesome bowel preparation quality such as patients with IBD remained to be confirmed. And lastly, as the study was conducted during the COVID-19 pandemic, almost 10% of the enrolled participants could not visit the hospital on their appointment dates. This factor may have affected the power of the study because the final sample size was slightly smaller than expected.

In conclusion, smartphone application-guided bowel preparation was associated with adequate bowel preparation similar with standard technique. However, the proportion of patients with excellent bowel preparation was significantly higher in the application group. Visualization of the right colon was better in the APP group. Therefore, education using smartphone applications may be an attractive option to improve bowel preparation outcomes in daily practice. Nonetheless, validating the utility of this application in patients with different backgrounds, more general population and larger sample sizes is required to confirm outcomes.

## Data availability statement

The raw data supporting the conclusions of this article will be made available by the authors, without undue reservation.

## Ethics statement

The studies involving humans were approved by the Ethics Committee of the Faculty of Medicine, Prince of Songkla University (REC No. 62-360-14-3). The studies were conducted in accordance with the local legislation and institutional requirements. The participants provided their written informed consent to participate in this study.

## Author contributions

TP: Conceptualization, Data curation, Formal analysis, Investigation, Methodology, Project administration, Resources, Software, Supervision, Validation, Visualization, Writing – original draft, Writing – review & editing. TK: Conceptualization, Data curation, Methodology, Project administration, Visualization, Writing – original draft, Writing – review & editing. AK: Data curation, Formal analysis, Writing – original draft. TW: Conceptualization, Data curation, Software, Writing – original draft. NN: Conceptualization, Data curation, Writing – original draft. NC: Conceptualization, Data curation, Writing – original draft. PS: Data curation, Formal analysis, Investigation, Supervision, Validation, Visualization, Writing – original draft, Writing – review & editing.
